# Who gets counted? Understanding low female death registration in India

**DOI:** 10.1371/journal.pone.0337224

**Published:** 2025-11-26

**Authors:** Sheetal Verma, Ritul Kamal, Laxmi Kant Dwivedi, Mrigesh Bhatia

**Affiliations:** 1 International Institute for Population Sciences (IIPS), Mumbai, Maharashtra, India; 2 Directorate of Census Operations, Uttar Pradesh, Lucknow, Uttar Pradesh, India; 3 Department of Health Policy, London School of Economics, London, United Kingdom; All India Institute of Medical Sciences - Gorakhpur, INDIA

## Abstract

**Background:**

Civil Registration and Vital Statistics (CRVS) systems are essential for governance, public health, and achieving SDGs however, gender gaps limit women’s access to rights and services, with under-registration of female vital events reinforcing their invisibility and distorting gender-responsive policies.

**Objectives:**

This study examines the drivers of low female death registration across India’s States and Union Territories, focusing on the roles of age, gender and wealth, with an aim to inform policies to strengthen CRVS systems and reduce gender disparities in vital statistics.

**Methods:**

The study utilizes data from NFHS-5 (2019–2021 for examining the factors associated with female death registration. Multivariable logistic regression models have been used to examine the impact of socio-economic and demographic factors on female death registration in India.

**Findings:**

The results highlight a significant gender gap in death registration (73% male vs. 64% female). The gap is widest in states like Bihar and Uttar Pradesh, while states like Kerala and Goa report near universal registration for both sexes. Gender gaps in housing and land ownership align with gaps in death registration, suggesting a strong association between asset ownership and registration. The results highlight association between wealth and death registration, with rates rising across quintiles for both sexes; however males consistently have higher registration rates. Among the poorest, the gap is widest which narrows down in the richest group. A gender gap in death registration persists across all age groups in India, beginning early, widening during working ages, and continuing into old age; while registration rates improve with age and wealth, women especially among the poorest remain under-registered, particularly in early and later life stages.

**Conclusions:**

Women in India encounter barriers to civil registration, and improving death registration demands systemic reforms, digital advancements, and community engagement Strengthening political commitment, collaboration, and public awareness will ensure inclusive, accurate records, enhancing CRVS for governance and policy.

## Introduction

Civil Registration and Vital Statistics (CRVS) systems provide accurate, legally certified data on births and deaths, forming the foundation for governance, policy-making, and social protection [1,2]. These systems are essential for tracking progress toward gender equality and the Sustainable Development Goals (SDGs) [3,4]. A well-functioning CRVS system generates vital statistics, including birth and death counts and medical causes of death, which are critical for public health decision-making [5,6]. However, in many developing countries, deficiencies in civil registration systems lead to significant data gaps, undermining efforts to monitor and safeguard citizens’ well-being. The failure to record vital events, particularly among marginalized populations, has been described as a “scandal of invisibility,” highlighting the urgent need for improvements in CRVS systems [7–9].

A persistent gender gap exists in CRVS systems worldwide, with women and girls often encountering barriers to birth, marriage, and death registration. Gender biases embedded in social and cultural norms, such as male-dominated property ownership and the higher likelihood of male deaths occurring in hospitals can intersect with national legal and death registration systems, reinforcing structural barriers and power imbalances that disadvantage women [10,11]. These barriers restrict the women’s access to essential services, including healthcare, education, social protection, and property rights [12]. Societal norms and systemic obstacles contribute to the under-registration of women’s vital events, reinforcing their invisibility in official records. While birth registration is fundamental to establishing legal identity and accessing services, death registration along with accurate determination of cause of death is equally crucial for recognizing individuals’ contributions, ensuring inheritance rights, and shaping gender-responsive public health policies. Reliable cause-of-death data are essential for understanding patterns of disease burden, identifying gender-specific health risks, and designing targeted interventions. The under-registration of female deaths further skews mortality statistics and impedes efforts to address gender-based health disparities [13].

Globally, cultural, socioeconomic, and legal challenges hinder women’s full participation in civil registration processes [3] and the lack of gender-disaggregated data exacerbates the difficulties in monitoring and addressing these inequalities. Research indicates that the coverage and completeness of CRVS data for female deaths is generally lower than that for male deaths, with under-registration disproportionately affecting disadvantaged populations; in addition, the quality of cause-of-death reporting also remains a significant concern. [4,14]. Without comprehensive death registration, marginalized groups remain excluded from critical policy interventions, increasing their vulnerability [3,4].

In India, the Registration of Births and Deaths Act, 1969 mandates the universal registration of births and deaths within 21 days of occurrence [2,15]. The country’s civil registration system operates through a hierarchical structure, with registrars at local, district, state, and national levels. Despite legal mandates, challenges persist in achieving full registration, particularly for female deaths. While birth registration rates for girls have improved in some regions, death registration remains inadequate due to perceived lack of benefits, systemic inefficiencies, and infrastructural limitations [2]. The absence of reliable death registration data hinders medical research and accurate mortality estimates, limiting India’s ability to implement evidence-based public health policies.

Although previous studies have documented lower death registration rates for females, a deeper examination of the factors influencing this disparity is necessary [1,5,12,16–18]. Additionally, gender gaps in education and awareness can indirectly lead to lower registration of female deaths [19]. The study is guided by a structural-legal and socio-economic utility framework, which views gender disparities in death registration as a product of both patriarchal social norms and the perceived legal/economic utility of registering a death (for inheritance, pensions, insurance, etc.), especially influenced by asset ownership and wealth status.

This study aims to explore the drivers of low female death registration in India across States and Union Territories, analysing how age, gender, and wealth impact registration rates. By identifying the underlying causes of gender disparities in death registration, the research seeks to provide insights that can inform policy measures to strengthen CRVS systems and promote gender equity in vital statistics.

## Materials and methods

### Data source

The paper has utilized the NFHS-5 data for studying the trends and factors associated with female death registration in India. The NFHS follows a consistent survey methodology nationwide, providing representative household samples across states and districts over multiple rounds. It offers reliable estimates on population, housing, fertility, family planning, maternal and child health, mortality, birth and death registration, nutrition, and morbidity across 28 states and 8 Union Territories. The study focuses on the data collected during NFHS-5 (2019−2021) [20]. NFHS-5, conducted in two phases between 2019 and 2021, covered all 707 districts in 28 states and 8 Union Territories. The sampling procedures and sample size selection for NFHS-5 is detailed in the NFHS-5 report [20]. The number of households interviewed in NFHS-5 were 636,699.

### Study variables

The primary outcome variable in this analysis is death registration. The NFHS in its latest round provides information on the number of deaths in a household since 2016. In the latest survey (2019−21), a question on death registration has been introduced which collects information from family members as “Death registered with the civil authority” [20]. The dependent variable for the study was “death registered with civil authority”, which was categorised into death registered as 1, otherwise 0.

In addition to death registration, several demographic and socio-economic variables are considered in the analysis to identify factors influencing death registration levels. These variables include the sex of the deceased (male or female), wealth index (as given in NFHS), religion of the head of the household (categorised as Hindu, Muslim, and Others), caste of the head of household (categorised as Scheduled Castes, Scheduled Tribe, and Others), health insurance (yes or no), place of residence (rural or urban), the structure of the household (nuclear or non-nuclear). The age of the deceased was categorised as less than 1 years, 1–4 years, 5–17 years, 18–59 years, 60–84 years and greater than 85 years. The age of the deceased was recoded into age 0–14 years, 15–59 years and 60 + years for the multivariable logistic regression analysis. The wealth index measures a household’s living standard based on asset ownership, housing materials, and access to water and sanitation. It is divided into five quintiles: poorest, poorer, middle, richer, and richest [21].

NFHS survey includes a question regarding house ownership, ‘Do you own this or any other house either alone or jointly with someone else?’ and the responses are categorised as Alone only; Jointly only; Both alone and jointly; and finally, does not own. Similarly, a question on ownership of land (agricultural or non-agricultural) was asked ‘Do you own this or any other house either alone or jointly with someone else?’ and the responses are categorised as Alone only; Jointly only; Both alone and jointly; and finally, does not own. The analysis utilizes the data to obtain estimates of house and land ownership by women aged 15–49 years and men aged 15–54 years [20]. The values for both variables were categorized as any ownership of land or housing and does not own.

### Ethics statement

The study used a secondary dataset which is freely available in the public domain. The survey agencies had obtained the prior consent from the respondents. The microdata are publicly available and no formal ethics approval was required to carry out research using this data source.

### Statistical analysis

Descriptive statistics are used to summarize the parameters associated with death registration by gender, age and socio-economic factors, accounting for the NFHS sampling design. The multivariable logistic regression analysis is used to identify the determinants of death registration, as dependent variable was binary (registered = 1 & not registered = 0). The model for multivariable logistic regression analysis was specified as


$$\text{logit}({\text{p}}_{\text{i}})=\beta_{\text{0}}+\beta_{\text{1}}{\text{X}}_{\text{1}\text{i}}+\beta_{\text{2}}{\text{X}}_{\text{2}\text{i}}+\ldots +\beta_{\text{k}}X_{\text{ki}},$$

where pᵢ denotes the probability that death i was registered, and X₁ᵢ … X_K_ᵢ represent covariates such as gender, wealth quintile, religion, caste, household structure, and place of residence. Odds ratios (OR = e^β) were estimated with 95% confidence intervals. Interaction terms between wealth and gender were included to assess differential effects. Logistic regression was chosen as it appropriately models binary outcomes, allows adjustment for multiple confounders, and yields interpretable effect measures relevant for policy analysis. The multivariate decomposition method was applied to quantify gender disparities in death registration. The observed gap between males and females was decomposed into the endowment component (E), reflecting differences in observable characteristics and the coefficient component (C), capturing differences in the influence of these characteristics. This approach allowed identification of structural versus behavioural factors underlying the gender gap. The value *p < 0.05* was considered to be statistically significant. All statistical analyses are conducted using STATA 18 [22], and the findings on birth registration levels are presented using Datawrapper [23], facilitating a comprehensive visualization of the data.

## Results

### 1. Gender disparity across States and Union Territories

**Fig 1:**
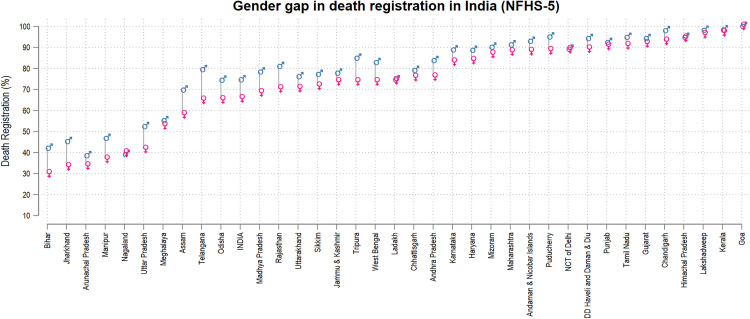
Gender gap in death registration in India (NFHS-5).

NFHS-5 data reveals a marked differential in the death registration rates of males and females. A total of 81,336 deaths were reported among the 636,699 households surveyed, out of which 46,917 (57.7%) were male and 34,419 (42.3%) were female. Of total male deaths, 73% (34,289) deaths were registered and of total female deaths, 64% (22,106) deaths were registered with the civil authorities, highlighting a marked gender disparity in death registration across the country. The national average also reflects this disparity with male death registration at 73% as compared to 64% for women. Male death registration rates are consistently higher than female rates, with the disparity being most pronounced in states like Bihar, Jharkhand, Uttar Pradesh, Arunachal Pradesh, Nagaland and Manipur. In contrast, states such as Goa, Kerala, and Lakshadweep show minimal gender differences, with registration rates exceeding 94% for both sexes (Fig 1).

### 2. Death registration by gender and housing & land ownership

NFHS collects data on housing and land ownership, key immovable assets in most states. The study considered only the immovable assets for analysis as the procedure for transfer/mutation of the same in government records is legally defined unlike ornaments, bullion, cattle and other forms of family wealth which change hands without any legal procedure.

**Fig 2:**
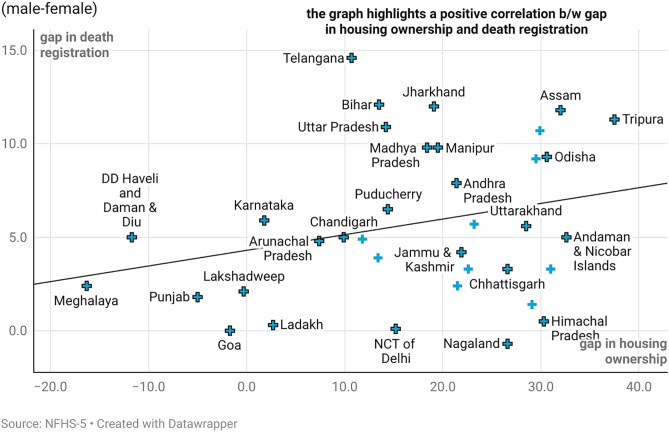
Gender gap in housing ownership and death registration.

**Fig 3:**
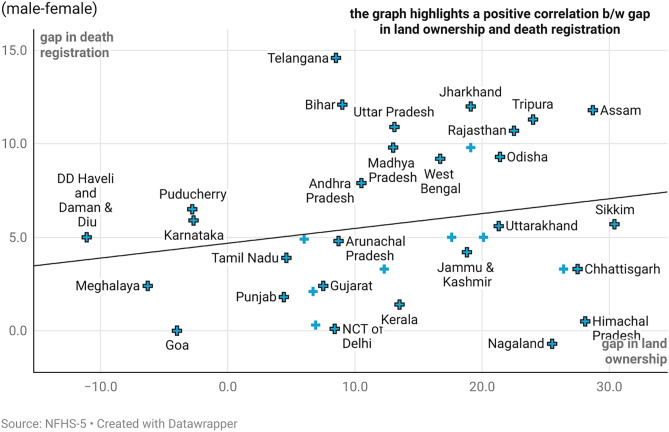
Gender gap in land ownership and death registration.

Fig 2 presents the gender gap in housing ownership and death registration while Fig 3 presents the gender gap in land ownership and death registration in India. The findings based on NFHS 5 data, reinforce this observation, indicating that death registration is closely linked to asset ownership, whether individual or joint (Fig 2 and 3).

On reading Figs 2–3 together, the states of Goa, Lakshadweep, Meghalaya, Dadra Nagar Haveli, Daman and Diu, Kerala and Tamil Nadu are low on gender gaps in both asset ownership and registration of deaths. Nearly, all the big States like Uttar Pradesh, Bihar, Rajasthan, Madhya Pradesh, Andhra Pradesh, Telangana, and even West Bengal and Assam exhibit gaps in both asset ownership and death registration of female vis a vis male (Figs 2 and 3). States like Nagaland and Himachal Pradesh show lower gap in death registration despite a huge gap in land ownership. The coverage and completeness of death registration in these States could explain the differential to a huge extent.

### 3. Age and wealth index differential in death registration

**Fig 4:**
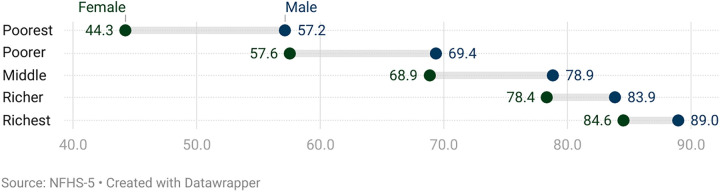
Death registration by gender and wealth index in India.

Fig 4 illustrates the gender gap in death registration across different wealth quintiles, revealing a clear socioeconomic gradient. Although death registration rates increase with wealth for both genders, death registration rates are consistently higher in males as compared to females. Among the poorest, male registration is 57%, while female registration lags behind at 44%, marking a substantial disparity. The gap persists across wealth categories, with male registration rates of 79% and female rates of 69% in the middle quintile. The gender gap in death registration narrows from 14% in the poorest to 4% in the richest wealth quintile, with male registration at 89% and female registration at 85% in the richest group—showing a slight but persistent disparity even among the wealthiest. (Fig 4).

**Fig 5:**
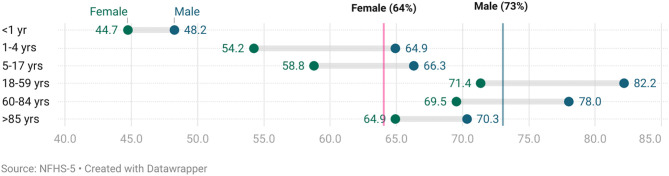
Death registration by age and gender in India.

Fig 5 presents death registration rates by age group and gender, highlighting a consistent gender gap across all age categories. Overall, male death registration (~73%) is significantly higher than female registration (~64%), reflecting a systemic disparity. The gap is evident from 0–4 age group, with male registration at 48.2% compared to 44.7% for females (Fig 5). This disparity widens during the early years (1–4 years: male 54.2%, female 64.9%) and persists across older age groups. Among the working age group (18–59 years), male registration reaches 82.2%, while female registration lags at 71.4%. The gender gap among the older age groups persists, however the registration rates for both male and female exceed the average highlighting possible association with economic benefits (related to inheritance, insurance, government compensation schemes etc) (Fig 5).

**Fig 6:**
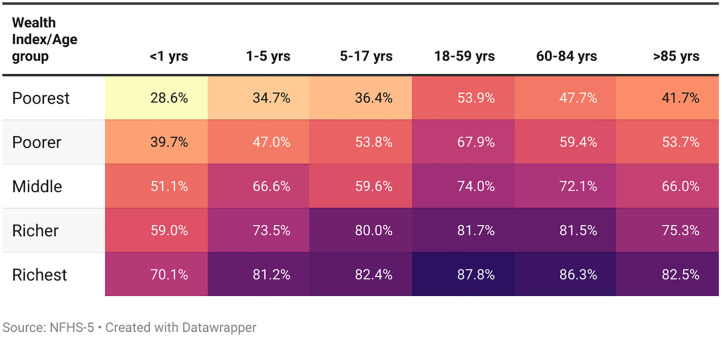
Female death registration by age group and wealth index.

Fig 6 presents the female death registration in India across different age groups stratified by wealth index categories. The figure highlights a strong positive association between wealth and death registration rates, with higher socioeconomic groups consistently exhibiting greater registration rates across all age groups. Among the poorest, registration rates are the lowest, beginning at 28.6% for infants (<1 year) and increasing to 41.7% for those above 85 years. In contrast, the richest group shows substantially higher rates, ranging from 70.1% in infancy to 82.5% in later years. Notably, within each wealth index group, registration rates generally increase with age, with highest rates being observed for the working cohort (18–59 years) (Fig 6).

### 4. Factors associated with death registration in India

Table 1 presents multivariable logistic regression analysis for identifying the determinants associated with death registration in India. The analysis identified several significant factors influencing death registration, with the effects most pronounced in the 15–59 and 60 + age groups. Wealth status emerged as a consistent predictor, where individuals from richer households had significantly higher odds of death registration compared to the poorest category. In the 15–59 age group, those from richer (OR: 3.89), richest (OR: 5.67), and middle (OR: 2.52) wealth quintiles were substantially more likely to register deaths. A similar pattern was observed in the 60 + group, with the richest households showing over five times higher odds (OR: 5.76) than the poorest. Female gender was also associated with lower odds of death registration in both the 15–59 (OR: 0.65) and 60+ (OR: 0.58) age groups. Notably, no significant interaction effects between wealth and gender were found in any age group, indicating that the positive effect of wealth on death registration operates similarly for both males and females. Religion significantly influenced death registration, with Muslims (OR: 0.65 in 15–59; OR: 0.60 in 60+) and other minority groups having lower odds compared to Hindus. Household structure mattered as well, with non-nuclear households showing reduced odds in the 15–59 (OR: 0.95) and 60+ (OR: 0.82) age groups. Furthermore, individuals in rural areas consistently had lower odds of death registration in these age groups (OR: 0.78 in 15–59; OR: 0.65 in 60+). Caste differences were largely non-significant, except among Scheduled Tribes in the 0–14 age group (OR: 2.99). In all, wealth, gender, religion, household type, and place of residence were key determinants of death registration, particularly affecting adults aged 15–59 and older adults aged 60 and above (Table 1).

**Table 1:** Factors associated with death registration.

Death registered with the civil authority	Age group 0–14	Age group 15–59	Age group 60+
	Odds Ratio (95% CI)	Odds Ratio (95% CI)	Odds Ratio (95% CI)
**Wealth Quantile**			
Poorest®			
Poorer	3.43 (1.49–7.88)**	1.59 (1.47–1.71)***	1.55 (1.36–1.78)***
Middle	2.08 (0.77–5.62)	2.52 (2.32–2.72)***	2.46 (2.13–2.84)***
Richer	7.22 (1.79–29.12)**	3.89 (3.55–4.26)***	3.28 (2.82–3.81)***
Richest	5.59 (1.02–30.50)*	5.67 (5.08–6.32)***	5.76 (4.82–6.89)***
**Gender**			
Male®			
Female	0.53 (0.24–1.15	0.65 (0.61–0.)***	0.58 (0.51–0.66)***
**Interaction**			
Wealth#Gender			
Poorer#Female	4.05 (1.23–13.33)*	0.94 (0.86–1.04)	1.05 (0.87–1.26)
Middle#Female	0.62 (0.14–2.59)	0.95 (0.85–1.05)	1.00 (0.82–1.22)
Richer#Female	0.41 (0.03–5.19)	1.00 (0.89–1.13)	0.95 (0.77–1.17)
Richest#Female	2.73 (0.27–27.61)	0.99 (0.86–1.14)	1.09 (0.85–1.39)
**Religion**			
Hindu®			
Muslim	0.45 (0.18–1.11)	0.65 (0.61–0.69)***	0.60 (0.53–0.67)***
Others	0.28 (0.11–0.68)**	0.73 (0.68–0.77)***	0.84 (0.75–0.94)**
**Caste**			
Schedule caste®			
Scheduled tribe	2.99 (1.29–6.89)*	1.03 (0.97–1.10)	1.01 (0.90–1.13)
Others	1.26 (0.65–2.47)	0.98 (0.93–1.02)	0.98 (0.89–1.06)
**Health insurance**			
No®			
Yes	0.39 (0.01–12.72)	1.12 (0.91–1.38)	1.32 (0.90–1.95)
**Household structure**			
Nuclear®			
Non-nuclear	1.16 (0.68–1.96)	0.95 (0.92–0.99)*	0.82 (0.76–0.88)***
**Place of Residence**			
Urban®			
Rural	0.46 (0.20–1.09)	0.78 (0.74–0.83)***	0.65 (0.59–0.72)***
_cons	0.82 (0.28–2.42)	1.13 (1.04–1.22)**	1.56 (1.33–1.82)***

* p < 0.05, ** p < 0.01, *** p < 0.001.

### 5. Explaining gender differences in death registration

Table 2 presents the results of decomposition analysis for the 15–59 and 60 + age groups, using gender (male and female) as the decomposing variable, and reveals a clear and consistent pattern: the observed difference in death registration between males and females is primarily driven by individual and household behavioural factors, reflected in the coefficient component of the models. The decomposition divides the gender gap into three parts: the endowment effect (E), capturing differences in observable characteristics between males and females; the coefficient effect (C), representing differences in how these characteristics influence registration and the residual effect (R), which accounts for the unexplained portion of the gap due to unobserved factors or model limitations.

**Table 2:** Decomposition of gender differences in death registration among adults aged 15–59 and 60 + years.

Reference group: Male; Comparison group: Female							
Age group	Effect	Coef.	SE	z	p-value	95% CI	Pct.
15–59	Endowment (E)	0.00007	0.00035	0.19	0.85	–0.0006–0.0008	0.1
	Coefficient (C)	0.0895	0.0038	23.8	<0.001	0.082–0.097	99.9
	Residual (R)	0.0896	0.0038	23.9	<0.001	0.082–0.097	—
60+	Endowment (E)	–0.0065	0.0006	–10.1	<0.001	–0.0078–0.0052	–7.1
	Coefficient (C)	0.0978	0.0065	15.1	<0.001	0.085–0.111	107.1
	Residual (R)	0.0913	0.0065	14.1	<0.001	0.079–0.104	—

Note: Coef. = Coefficient; SE = Standard Error; CI = Confidence Interval; Pct. = Percentage contribution.

In the 15–59 age group, the mean death registration was higher among males (0.7274) than females (0.6382). The decomposition results revealed that key behavioural factors contributed significantly to this difference, with coefficients of 0.0895 (p < 0.001) accounting for nearly 99.9% of the explained variation in death registration between the genders. Similarly, in the 60 + age group, males had a higher mean death registration rate (0.7493) compared to females (0.6601). Here again, behavioural factors made substantial contributions to the gender difference with coefficients of 0.0978 (p < 0.001) explaining 107.1% of the difference, respectively (Table 2).

## Discussion

While India has made significant progress in birth registration, largely driven by the rise of institutional deliveries and financial incentive schemes such as the Janani Suraksha Yojana [24], death registration continues to face systemic challenges. Unlike births, which increasingly take place in hospitals, deaths remain a dispersed socio-cultural event, often occurring at home, in rural areas, or in communities where formal reporting structures are weak [12]. The lack of institutionalization and economic incentives makes registering deaths a complex exercise. Unlike birth registration, which is often linked to identity documents and welfare programs, death registration is frequently overlooked unless it has immediate legal or financial implications, which results in underreporting, particularly for marginalized groups, including women, children, and the poor.

The analysis clearly indicates that the gender gap in death registration, particularly in the 15–59 and 60 + age groups, is overwhelmingly explained by individual and household behavioural factors such as awareness, attitudes towards formal death reporting, and household decision-making pattern. The finding underscores the importance of addressing behavioural barriers and promoting awareness initiatives to improve death registration rates, especially among females in these age groups. In addition to this, the decomposition analysis results identified several individual-level factors significantly associated with death registration, particularly in the 15–59 and 60 + age groups. Wealth status showed a clear positive association with death registration, with individuals from richer and richest households being substantially more likely to register deaths than those from the poorest group. Male gender was consistently associated with higher levels of death registration across these age groups compared to females. Religion also played an important role. Furthermore, individuals from non-nuclear households and those residing in rural areas had positive contribution of registering deaths. These findings confirm that death registration is strongly influenced by socio-economic and household-level behavioural factors. 

The analytical approach of the study is both rigorous and well-aligned with the research objectives. Multivariable logistic regression models were employed to analyze nationally representative NFHS-5 microdata, controlling for key socio-demographic confounders. To capture intersectional patterns of disadvantage, stratified analyses by wealth and age groups were also conducted. This combined strategy enhances statistical robustness and allows for a nuanced interpretation of gender disparities in death registration within the Indian context.

Death registration plays a crucial role in public health monitoring, serving as the vital component for measuring maternal and infant mortality—both of which are key indicators for Sustainable Development Goals (SDGs). Although the Sample Registration System provides state-level estimates of IMR and MMR, strengthening the accuracy and completeness of death records can improve the precision of these estimates and enable more granular and targeted health interventions [22]. India’s commitment to SDG 3.1, which aims to reduce maternal mortality to less than 70 per 100,000 live births by 2030, depends on reliable death registration data [25,26]. Strengthening female death registration is particularly important for improving maternal health policies, identifying high-risk areas, and implementing evidence-based interventions to reduce preventable deaths.

While there is a documented gender gap in death registration, attributing this disparity solely to gender bias would be an oversimplification. The narrowing of the gender gap in the working-age cohort (18–59 years) suggests that economic and legal factors also play a crucial role. Deaths in this age group are often associated with employment-related benefits, insurance claims, or compensation schemes, which may drive families to register them irrespective of gender. The finding of the study challenge the assumption that cultural biases alone account for the under-registration of female deaths and underscores the need for death registration to be undertaken on a *suo-moto* basis by state-appointed registrars. This is particularly crucial for women without asset ownership or participation in the formal economy.

Surprisingly, despite the massive outreach of health and community workers in the country, most of whom are designated as ‘informants’ and despite the continued emphasis on tracking infant mortality (0–1 years) and child mortality (1–5 years), the fact that 50% of deaths in this age cohort are unregistered, shows a major gap in our health policy implementation. [18]. While the rate of death registration is indeed around 51% in the 0–4 age bracket, even in the case of small children and the under 18 population, the death of a male household member shows a higher registration trend compared to females in every comparable age cohort. This is a clear indication of the patrilineal inheritance laws where any male death changes the succession chart and thus needs to be registered unlike females in every age bracket who are less likely to claim/own immovable assets at any point of time in their lives. Social stigma surrounding infant mortality, combined with inadequate awareness of the importance of death registration, exacerbates the underreporting of female child deaths [2,18,27,28]. Addressing these gaps requires targeted interventions at the community level, particularly through frontline health workers such as Anganwadi workers, ASHAs, and municipal registrars.

Ownership of assets and wealth has also exhibited a strong relationship with death registration, as highlighted in the case of matrilineal societies such as Kerala, Goa, Lakshadweep and parts of the Northeast [29–33]. NFHS-5 data highlights that women in India own significantly fewer assets than men, which affects the perceived necessity of registering their deaths [34]. Households with greater economic stability and legal awareness are more likely to register deaths, as exhibited in the highest wealth quintile, where gender disparities in registration are minimal [18,28]. Strengthening female property rights and linking death registration to social benefits, such as pensions and inheritance claims, could serve as effective policy measures to improve registration rates. Deaths of women who own land or property are more likely to be registered compared to those who do not. Family members often ensure the registration of female deaths to facilitate the smooth transfer of property and assets within the household. Since official documentation is required for inheritance and other legal benefits, registering deaths of female members who own land or property becomes a necessary step.

Overall, improving death registration in India requires a multi-pronged strategy that integrates legal reforms, digital innovations, and grassroots-level awareness campaigns. The recent amendments to the Registration of Births and Deaths (RBD) Act, 2023 [15,35] provide an opportunity to strengthen reporting mechanisms and streamline processes. Region-specific and gender-sensitive interventions are essential for enhancing women’s access, particularly within rural contexts. Empowering female community health workers to report deaths is a critical measure that can elevate registration rates and reduce gender bias, especially in areas with limited access to registration facilities [19]. Linking registration with social protection schemes, digitizing records, and leveraging mobile-based registration platforms can help bridge existing gaps. Ultimately, ensuring comprehensive death registration, built on *suo-moto* registration of all vital events particularly for women and disadvantaged groups, is crucial for building an inclusive and equitable Civil Registration and Vital Statistics (CRVS) system that serves as a cornerstone for governance, policy-making, and social justice.

## Conclusion

Women and girls play a central role throughout the life course, often serving as primary caregivers during both birth and end-of-life care. Notably, many births and deaths occur in the presence of healthcare workers, a majority of whom are women. However, they continue to face significant challenges in accessing civil registration. Improving death registration in India requires a comprehensive strategy that combines systemic reforms, digital advancements, and community participation. Strengthening political commitment, fostering interdepartmental collaboration and raising public awareness—alongside digitization and centralized databases will ensure accurate, inclusive, and accessible records, transforming CRVS into a powerful tool for governance, policy formulation, and social well-being.

## Limitations

The study used secondary data from NFHS-5. Despite a large sample size, self-reported data may be affected by recall bias.
